# Replisome bypass of a protein-based R-loop block by Pif1

**DOI:** 10.1073/pnas.2020189117

**Published:** 2020-11-16

**Authors:** Grant D. Schauer, Lisanne M. Spenkelink, Jacob S. Lewis, Olga Yurieva, Stefan H. Mueller, Antoine M. van Oijen, Michael E. O’Donnell

**Affiliations:** ^a^Department of Biochemistry and Molecular Biology, Colorado State University, Fort Collins, CO 80523;; ^b^Molecular Horizons and School of Chemistry and Molecular Bioscience, University of Wollongong, Wollongong, NSW 2522, Australia;; ^c^Illawarra Health & Medical Research Institute, Wollongong, NSW 2522, Australia;; ^d^HHMI, Rockefeller University, New York, NY 10065;; ^e^Laboratory of DNA Replication, Rockefeller University, New York, NY 10065

**Keywords:** DNA replication, single-molecule, replisome, Pif1, replication bypass

## Abstract

Efficient and faithful replication of the genome is essential to maintain genome stability. Replication is carried out by a multiprotein complex called the replisome, which encounters numerous obstacles to its progression. Failure to bypass these obstacles results in genome instability and may facilitate errors leading to disease. Cells use accessory helicases that help the replisome bypass difficult barriers. All eukaryotes contain the accessory helicase Pif1, which tracks in a 5′–3′ direction on single-stranded DNA and plays a role in genome maintenance processes. Here, we reveal a previously unknown role for Pif1 in replication barrier bypass. We use an in vitro reconstituted *Saccharomyces cerevisiae* replisome to demonstrate that Pif1 enables the replisome to bypass an inactive (i.e., dead) Cas9 (dCas9) R-loop barrier. Interestingly, dCas9 R-loops targeted to either strand are bypassed with similar efficiency. Furthermore, we employed a single-molecule fluorescence visualization technique to show that Pif1 facilitates this bypass by enabling the simultaneous removal of the dCas9 protein and the R-loop. We propose that Pif1 is a general displacement helicase for replication bypass of both R-loops and protein blocks.

## Significance

The replisome machine that duplicates the DNA genome encounters a variety of blocks to replication, such as DNA bound proteins, R-loops, and DNA lesions. Studies in bacterial systems demonstrate that replisome advance through blocks is facilitated by an accessory monomeric helicase that acts on the opposite strand from the replicative hexameric helicase. This report examines this issue in a eukaryotic system, using Pif1, a monomeric helicase that acts on the opposite strand from the replicative CMG helicase. The report shows that an inactive “dead” Cas9 (dCas9) protein R-loop block arrests the replisome, but Pif1 enables replisome bypass of the dCas9 R-loop block. Hence, use of monomeric helicases may have evolved to aid replisome bypass of protein-DNA and protein-bound R-loop blocks.

Efficient and faithful replication of the genome is essential to maintain genome stability and is carried out by a multiprotein complex called the replisome (1–4). There are numerous obstacles to progression of the replisome during the process of chromosome duplication. These obstacles include RNA-DNA hybrids (R-loops), DNA secondary structures, transcribing RNA polymerases, and other tightly bound proteins (5–9). Failure to bypass these barriers may result in genome instability, which can lead to cellular abnormalities and genetic disease. Cells contain various accessory helicases that help the replisome bypass these difficult barriers (10–20). A subset of these helicases act on the opposite strand of the replicative helicase (1, 2, 14, 19).

All eukaryotes contain an accessory helicase, Pif1, which tracks in a 5′–3′ direction on single-stranded DNA (ssDNA) (11–16). Pif1 is important in pathways such as Okazaki-fragment processing and break-induced repair that require the removal of DNA-binding proteins as well as potential displacement of R-loops (11–13, 21, 15–18, 22–25). Genetic studies and immunoprecipitation pull-down assays indicate that Pif1 interacts with PCNA (the DNA sliding clamp), Pol ε (the leading-strand polymerase), the MCMs (the motor subunits of the replicative helicase CMG), and RPA (the single-stranded DNA-binding protein) (15, 26, 27). Pif1 activity in break-induced repair strongly depends on its interaction with PCNA (26). These interactions with replisomal components suggest that Pif1 could interact with the replisome during replication. In *Escherichia coli*, the replicative helicase is the DnaB homohexamer that encircles the lagging strand and moves in a 5′–3′ direction (20). *E. coli* accessory helicases include the monomeric UvrD (helicase II) and Rep, which move in the 3′–5′ direction and operate on the opposite strand from the DnaB hexamer. It is known that these monomeric helicases promote the bypass of barriers during replication such as stalled RNA polymerases (5). The eukaryotic replicative helicase is the 11-subunit CMG (Cdc45, Mcm2–7, GINS) and tracks in the 3′–5′ direction, opposite to the direction of Pif1 (25, 28). Once activated by Mcm10, the MCM motor domains of CMG encircle the leading strand (29–32). We hypothesized that, similar to UvrD and Rep in *E. coli*, Pif1 interacts with the replisome tracking in the opposite direction to enable bypass of replication obstacles.

In this report, we use an in vitro reconstituted *Saccharomyces cerevisiae* replisome to study the role of Pif1 in bypass of a “dead” Cas9 (dCas9), which is a Cas9 protein that is deactivated in DNA cleavage but otherwise fully functional in DNA binding. As with Cas9, dCas9 is a single-turnover enzyme that can be programmed with a guide RNA (gRNA) to target either strand. The dCas9–gRNA complex forms a roadblock consisting of an R-loop and a tightly bound protein (dCas9), a construct that is similar to a stalled RNA polymerase. This roadblock (hereafter dCas9 R-loop) arrests replisomes independent of whether the dCas9 R-loop is targeted to the leading or lagging strand (30). Besides its utility due to its programmable nature (33), the use of the dCas9 R-loop allows us to answer several mechanistic questions. For example, the ability to program the dCas9 R-loop block to any specific sequence enables us to observe whether block removal is different depending on whether the block is on the leading or lagging strand. Furthermore, the inner diameter of CMG can accommodate double-stranded DNA (dsDNA) and possibly an R-loop, but not a dCas9 protein. Using the dCas9 R-loop block allows us to determine the fate of each of its components.

Here, we report that Pif1 enables the bypass of the dCas9 R-loop by the replisome. Interestingly, dCas9 R-loops targeted to either the leading or lagging strand are bypassed with similar efficiency. In addition, the PCNA clamp is not required for bypass of the block, indicating that Pif1 does not need to interact with PCNA during bypass of the block. We used a single-molecule fluorescence imaging to show that both the dCas9 and the R-loop are displaced as an intact nucleoprotein complex. We propose that Pif1 is a general displacement helicase for replication bypass of both R-loops and protein blocks.

## Results

In this study, we used a dCas9 R-loop block to determine how Pif1 may assist the replisome in overcoming a protein-bound RNA-loop barrier to replication fork progression. We studied the effect of Pif1 on reconstituted replisomes using both ensemble and single-molecule methods. To ensure we are not observing the processive Pif1–Pol δ–PCNA strand-displacement complex that has been reported previously (26), we initially excluded Pol δ from ensemble reactions, focusing on CMG–Pol ε mediated leading-strand progression. We then moved to single-molecule studies containing all replisomal factors including Pol δ.

### Pif1 Acts Distributively to Enable Replisome Bypass of a dCas9 R-Loop Block.

**Fig. 1.**
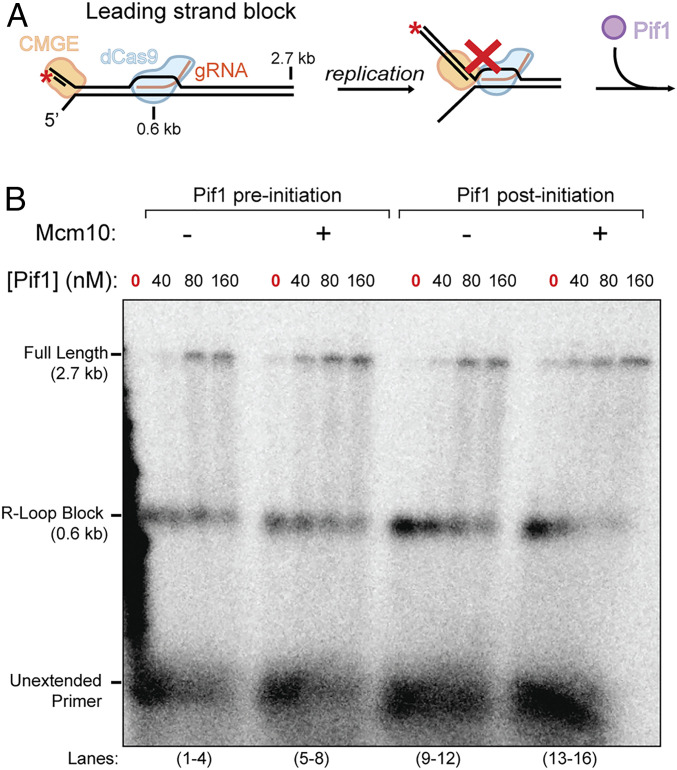
Pif1 facilitates replisome bypass of a leading-strand dCas9 block. (*A*) Illustration of the primed forked DNA and dCas9 R-loop block. The ^32^P-labeling position of the primer is denoted as a red asterisk. The CMG–Pol ε complex is labeled as CMGE. (*B*) Alkaline agarose gel analysis of the products. Where indicated, replication reactions were carried out with Mcm10 present in the initial incubation (*Methods*).

**Fig. 2.**
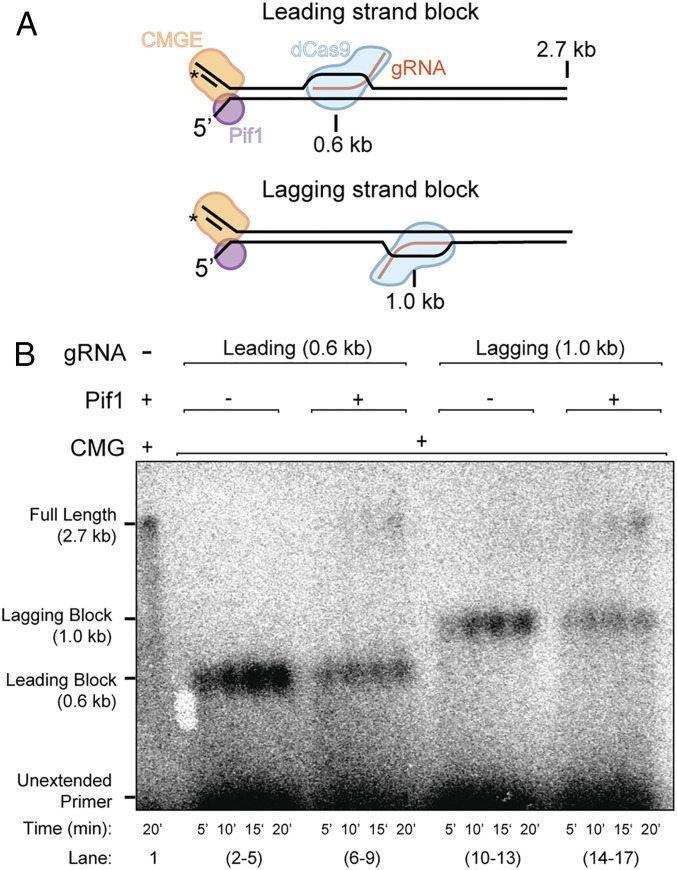
Pif1 enables fork progression through a dCas9 R-loop block on either the leading or lagging strand. (*A*) Illustration of the 3-kb forked DNA with either a leading or lagging strand dCas9 R-loop block. (*B*) Alkaline agarose gel of the results with or without dCas9 having a gRNA directed against the leading or lagging strand. Experiment staged as described in *Methods*, with the exception that 40 nM Pif1 was added for 5 min after the initial incubation with CMG, dCas9, and gRNA. Time courses include the first 5-min run-up to the block before the with or without Pif1 step.

We used an ensemble replication assay to visualize products generated through leading-strand synthesis. We assembled a forked DNA substrate by ligating a synthetic replication fork to a linearized 2.7-kb plasmid and primed it on the leading strand with a 5′ ^32^P-labeled DNA oligonucleotide, illustrated in Fig. 1*A*. To establish leading-strand replication by the CMG–Pol ε (CMGE) complex (34), CMG was added for 5 min in the absence of nucleotide. Pol ε, PCNA, and RFC along with dNTPs were added subsequently and incubated for 5 min. The reaction was initiated upon addition of ATP and RPA (see *Materials and Methods* for details). Products were separated on an agarose gel, and extension of the leading strand ^32^P-primer was visualized as previously described (35). To introduce a dCas9 R-loop block, we designed a gRNA positioned on the leading strand ∼0.6 kb away from the replication fork (Fig. 1*A*). The gRNA and dCas9 were preincubated with the template DNA before addition of CMG. Consistent with previous studies, the dCas9 R-loop is shown to be a potent roadblock to replication (Fig. 1*B*, lane 1) (33). A control reaction lacking the block is shown later in Fig. 2.

Next, we tested whether Pif1 helicase could help the replisome bypass a dCas9 R-loop block on the leading strand. Given that Pif1 has known interactions with replisome components, it is possible that Pif1 could travel with the fork. Therefore, we hypothesized that the efficiency of bypass would be optimal if Pif1 was preincubated with the replisome before initiation. To test this, we first preincubated Pif1 at the same time as CMG, followed by Pol ε addition. In the presence of Pif1, we observed bypass of a leading strand-dCas9 R-loop block. The efficiency of bypass is dependent on the concentration of Pif1, suggesting that Pif1 activity is distributive (Fig. 1*B*, lanes 1–8). Next, we tested whether Pif1 could rescue a stalled replisome. When we added Pif1 after allowing synthesis up to the block for 5 min, we observed bypass of the dCas9 R-loop block (Fig. 1*B*, lanes 9–16). The results were similar to the R-loop bypass activity observed when Pif1 was preincubated with replication components (Fig. 1*B*; lanes 1–8). This similarity suggests that Pif1 is able to rescue stalled replisomes in trans, rather than needing to first preassemble into the replisome, and is consistent with distributive action of Pif1. Importantly, we found that Pif1 alone does not displace the dCas9 R-loop (*SI Appendix*, Fig. S1), suggesting Pif1 operates in conjunction with the replisome. We also tested the effect of Mcm10, a replisome component that stimulates fork progression about 1.5-fold and that has been shown to help the replisome bypass nucleoprotein blocks (32, 36). Inclusion of Mcm10 stimulates bypass of the dCas9 R-loop by Pif1 (Fig. 1*B*, lanes 5–8) to a similar extent as Mcm10 stimulation of a replisome lacking a block (32, 36), suggesting that Pif1 acts independently of Mcm10. Thus, in the context of replication fork progression, Pif1 is acting outside of the previously described block-bypass mechanisms to enable bypass of a leading-strand R-loop block.

### Pif1 Facilitates Bypass of Leading and Lagging-Strand dCas9 R-Loops Blocks.

Next, we compared results using a dCas9 R-loop block having a gRNA targeted to either the leading or lagging strand, as illustrated in Fig. 2*A*. We used a suboptimal concentration of Pif1 (40 nM), in order to more clearly detect if there might be a difference in bypass for the two different blocks. In lane 1 in Fig. 2*B*, we carried out replication using Pif1 and the replisome factors on unblocked DNA and followed bypass by alkaline agarose gel electrophoresis. Interestingly, the results in Fig. 2*B* show that a dCas9 R-loop block is bypassed with similar efficiency with an R-loop targeted to the leading or lagging strand.

### PCNA Does Not Enhance Pif1 Function in Bypass of the dCas9 R-Loop Block on 3-kb DNA.

**Fig. 3.**
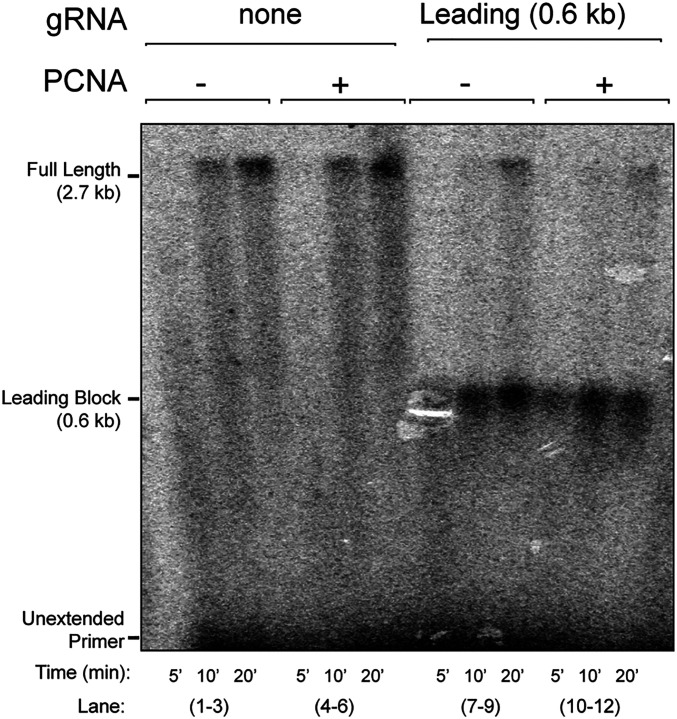
PCNA is not required for Pif1 mediated bypass of a leading-strand dCas9 block. Replication time courses in the absence (lanes 1–6) or presence (lanes 7–12) of a leading-strand dCas9 RNA-loop block, with or without the addition of PCNA, as indicated.

Pif1 is known to interact with PCNA, and this interaction is important to the function of Pif1 with Pol δ in break-induced replication repair (26, 27). Hence, we next probed whether PCNA is required to observe replisome bypass of the dCas9 R-loop (Fig. 3). As a control, lanes 1–6 show products from time-course measurements using unblocked 3-kb forked DNA. Full-length products are observed, even without PCNA as we reported previously (37). On longer DNA templates, PCNA is known to stimulate fork progression, consistent with Pol ε being held to DNA by both CMG (34) and PCNA (38). We presume that PCNA is normally required for leading-strand replication, but 3 kb is perhaps too short a distance to observe the effect of PCNA on replisome efficiency, as we suggested earlier (39). Lanes 7–12 in Fig. 3 show that Pif1-dependent bypass is similar in the presence or absence of PCNA. We observed a similar independence of PCNA clamp for bypass of a lagging-strand block (*SI Appendix*, Fig. S2). We also examined the effect of PCNA on dCas9 bypass in the presence of the fork-protection complex, Mrc1–Tof1–Csm3 (MTC), a complex known to stimulate fork progression (32, 36, 38, 40). However, addition of MTC did not enable Pif1 and PCNA to bypass the dCas9 block (*SI Appendix*, Fig. S3). Hence, Pif1 does not require interaction with PCNA to facilitate replisome bypass of the dCas9 R-loop block. This suggests that the action of Pif1 in promoting replisome bypass of a dCas9 R-loop may be distinct from its action with Pol δ–PCNA in break-induced repair (*Discussion*).

### Single-Molecule Studies Show Pif1 and the Replisome Act Together to Bypass a dCas9 Block.

**Fig. 4.**
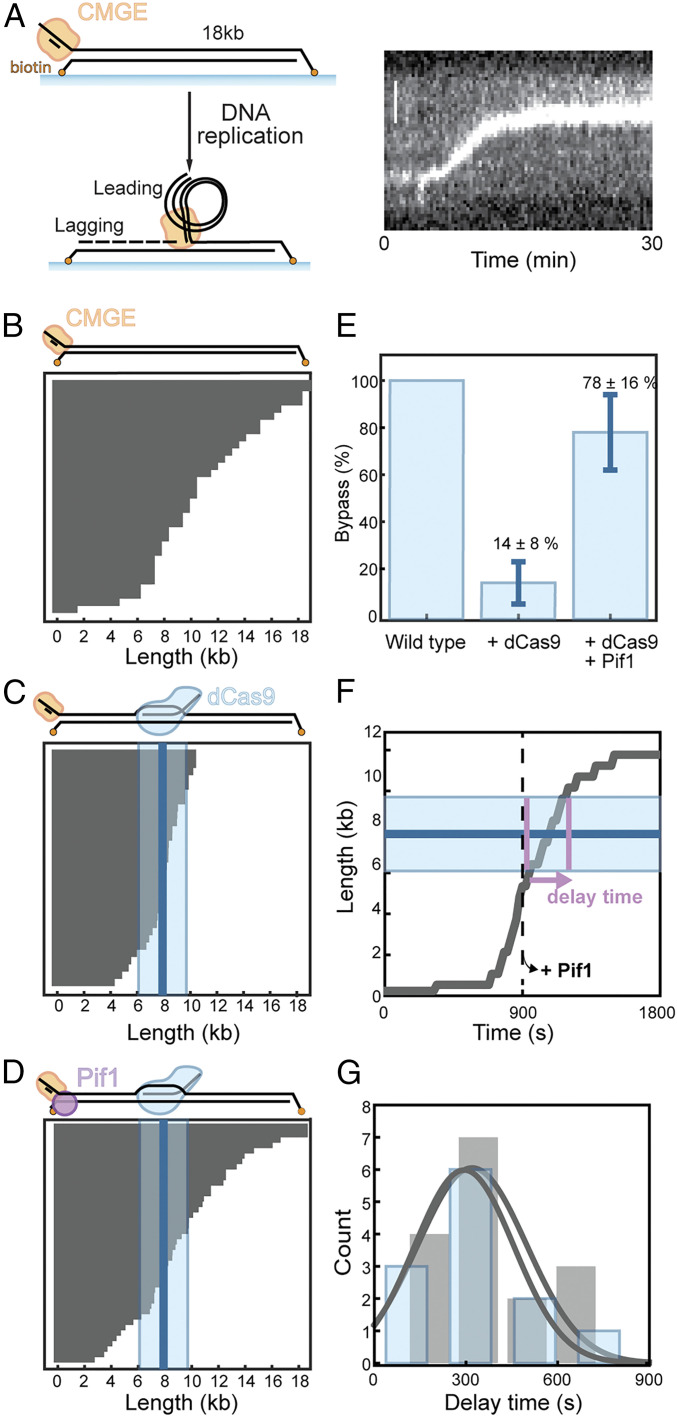
Single-molecule examination of Pif1-dependent replisome bypass of a dCas9 blocked site. (*A*) Schematic representation of the single-molecule assay (described in text) and a representative kymograph. Leading-strand DNA (stained with SYTOX Orange) accumulates along with a progressing replication fork. (*B*) Replication product lengths for replication without a dCas9 block. Every gray line represents the length of a single molecule. (*C*) Replication product lengths for replication in the presence of the dCas9 block bound to the leading strand. The blue line represents the average position of the dCas9 block. The SD is indicated by the light blue area. (*D*) Replication product lengths for replication in the presence of the dCas9 block where Pif1 was added after 15 min. (*E*) Efficiency of bypass of the dCas9 block for the experiments in *B*–*D* normalized to the experiment without block. We define a block as bypassed when a replisome moves past the average position of the block + SD (9.8 kb). For the reaction without dCas9 block (*A*), we define the “bypass” efficiency as the number of replisomes that move past 9.8 kb. The error bars represent the SEM from three experiments. (*F*) Representative single-molecule trajectory showing the position of the replisome as a function of time (gray line). The blue line represents the position of the dCas9 block. The SD is indicated by the light blue area. Pif1 was added after 15 min (indicated by the dashed line). We define the delay time as the time it takes the replisome to move through the area indicated by the SD of the block position.(indicated by the pink arrow). (*G*) Histograms of the delay time for replication without dCas9 block (gray) and replication in which a leading strand block is resolved by Pif1 (blue). Again, for the reaction without dCas9 block, we define “bypass” when replisomes move past 9.8 kb. Gaussian fits (represented by the gray lines) give average delay times of 294 ± 137 s (mean ± SEM) for replication without block and 320 ± 150 s (mean ± SEM) for blocked replication resolved by Pif1.

Our ensemble assays show that Pif1 enables bypass of a dCas9 R-loop block but cannot decipher the mechanistic details of the fate of the dCas9 R-loop block upon bypass. Hence, we turned to single-molecule studies of this reaction. We assembled linear dsDNA molecules (18.3 kb in length) in a microfluidic flow cell placed onto a fluorescence microscope (41). The DNA is stretched and attached to the surface at both ends (Fig. 4*A*). A synthetic replication fork at one end of the DNA enables assembly of the replisome at one end (Fig. 4*A*). DNA synthesis was initiated by loading CMG and Mcm10 onto the template followed by the introduction of Ctf4; MTC; PCNA; RFC; RPA; DNA polymerases Pol α, Pol δ, Pol ε; and the four dNTPs and four rNTPs (41). DNA is visualized using a fluorescent DNA stain, and the assay is carried out in the absence of flow, relying on the extended state in which the DNA substrates are coupled to the surface. The leading-strand product that is extruded from the replisome collapses into a coil growing in size and intensity as the replisome progresses through the template (Fig. 4*A*). This reconstituted replisome is able to synthesize both the leading and the lagging strands and travels at rates that match previously measured physiological rates (41). By tracking the position of the leading-strand spot, we can determine the final product length for each individual DNA template (Fig. 4*B*). The finite processivity of the replication reaction gives rise to a distribution of product lengths that decays exponentially, with short products more likely than long ones. As such, some replication products are truncated before reaching the end of the DNA template.

Next, we introduced a fluorescently labeled dCas9 R-loop block. In contrast to our ensemble leading-strand assays, our single-molecule imaging experiments contain Pol α, Pol δ, and MTC. We repeated our ensemble assays in the presence of these factors to show that these factors do not enable the replisome to bypass the dCas9 R-loop block in the absence of Pif1 (*SI Appendix*, Fig. S4). Fig. 4*C* shows the replication product lengths in the presence of the dCas9 R-loop block as measured in the single-molecule assay. The position of the labeled dCas9-gRNA block was in good agreement with the expected position. The average localization of fluorescent dCas9 on DNA is represented as a solid line, and the surrounding box represents the SD (Fig. 4*C*). Due to the single-exponential distribution of product lengths, some replisomes do not make it to the dCas9 R-loop block. Similar to our observations in ensemble experiments (e.g., Figs. 1–3), the replisome without Pif1 is inefficient at proceeding past the block in our single-molecule assay (Fig. 4*C*). In the presence of Pif1, however, bypass is clearly observed, well beyond the level of uncertainly of the block position (Fig. 4*D*). In these assays, Pif1 is added 15 min after starting the replication reactions. Quantification shows 78% bypass of the dCas9 block by this approach (Fig. 4*E*). When we added Pif1 by itself, without the presence of any replisome components, we do not observe any block removal (*SI Appendix*, Fig. S1). This lack of removal shows that Pif1 is unable to remove the block by itself and, therefore, works in concert with the replisome.

Examination of single-molecule trajectories indicate a short delay time for replisome bypass of the dCas9 by Pif1 (Fig. 4*G*). We define the delay time as the time it takes the replisome to traverse through the entire ∼4-kb window representing the average dCas9 position and its associated uncertainty. We measure a delay time of 320 ± 150 s (mean ± SEM), which is within the experimental error for the delay time measured without a dCas9 block (294 ± 137 s [mean ± SEM]). Thus, Pif1 enables replication through an R-loop barrier in a timeframe that is consistent with unimpeded replication. We note that it is still possible that Pif1-assisted resolution of dCas9 results in a delay shorter than the resolution of our assay.

### Fate of dCas9 and gRNA during Replisome Bypass.

**Fig. 5.**
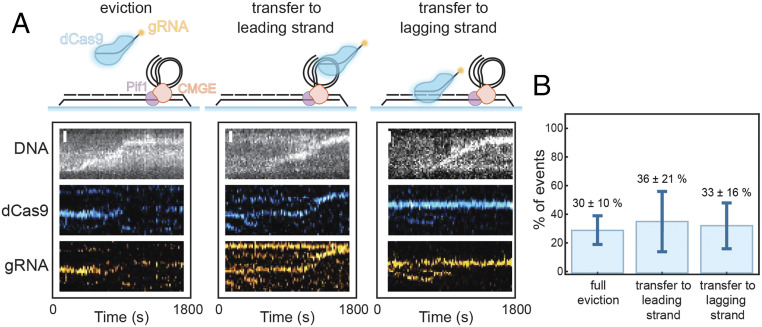
Single-molecule analysis of the fate of the dCas9 and R-loop components of the block. (*A*) Schematic illustrations and representative kymographs showing the DNA (*Top*, gray), fluorescently labeled dCas9 (*Middle*, blue), and fluorescently labeled gRNA (*Bottom*, orange). Three possible outcomes are observed: The block is evicted (*Left*), the block is transferred to the leading strand (*Center*), or the block is transferred to the lagging strand (*Right*). (*B*) Quantification of the observed frequency for the different behaviors outline in *A*. The error bars represent the SEM from three separate experiments.

To determine the fate of the dCas9 protein and the R-loop during replisome bypass, we used fluorescently labeled gRNA and dCas9 directed to the leading strand. SYTOX Orange stain was used to visualize duplex DNA. We observed three types of behaviors when the replisome encounters the block: 1) The signals of the dCas9 and the R-loop disappear (Fig. 5 *A*, *Left*); 2) dCas9 and the R-loop move with the fork, which indicates a transfer to the leading strand (Fig. 5 *A*, *Center*); and 3) dCas9 and the R-loop are transferred to the lagging strand, behind the fork (Fig. 5 *A*, *Right*). It is important to note that in all three of these outcomes, the Cas9 and gRNA signals behave the same. The results showed that both the R-loop RNA and dCas9 protein were evicted about 30% of the time (Fig. 5*B*). Interestingly, the R-loop and the dCas9 protein went to either the leading or lagging daughter strands with a similar frequency to their eviction (Fig. 5*B*). This observation implies that the dCas9 R-loop is removed as a whole and is able to rapidly rebind DNA. When the dCas9 R-loop is evicted, the chance of rebinding to the DNA is high. Its rapid search for a match to the gRNA results in rebinding of the dCas9 complex to one of the newly synthesized daughter strands. Consistent with both leading- and lagging-strand duplexes being synthesized in this experiment, we observed an equal distribution of dCas9-gRNA to both strands (Fig. 5*B*).

## Discussion

We find here that Pif1 can function in concert with the replisome to eject and bypass a dCas9 R-loop block (42). R-loops are thought to occur at transfer RNA (tRNA) gene clusters and other highly transcribed genes in vivo. Such R-loops are known to be difficult barriers to replication, and their presence may lead to genome instability (16, 18). Previously, Pif1 and Rrm3 helicases were shown to redundantly bypass a replication fork arrested at actively transcribing tRNA genes, in cooperation with the strand-displacement activity of Pol δ on the lagging strand (24). Here, we demonstrate that Pif1 is additionally capable of enabling the bypass of protein R-loop roadblocks on either the lagging or leading strands in the absence of Pol δ. The efficiency of displacement was detectably higher on a dCas9 R-loop directed to the lagging strand (*SI Appendix*, Fig. S3). Since the DNA–RNA hybrid of dCas9 is presented to Pif1 when targeted to the lagging strand (Fig. 2*A*), it is possible that the DNA–RNA helicase activity of Pif1 assists R-loop displacement in this configuration. Although with slightly lower efficiency, Pif1 still enables robust displacement of a leading-strand R-loop, which lacks a DNA–RNA duplex that is accessible to the replication fork. Indeed, Pif1 was recently shown to translocate on ssDNA at ∼140 nt/s (43), which may be sufficient to evict even strong R-loop blocks. Furthermore, we show here that neither Mcm10, the fork-protection complex MTC, nor PCNA were required for Pif1 to assist CMGE in displacing the dCas9 R-loop barrier. This unassisted and strand-unbiased R-loop displacement activity demonstrates yet another versatility of Pif1 in assisting regular replication-fork progression.

It is interesting to note that the current study shows PCNA is not needed for Pif1 action in promoting replisome bypass of a dCas9 block. This observation contrasts with studies that show Pif1 binds to PCNA and may do so for processive Pif1–Pol δ mediated break-induced replication repair (15, 26, 27). In the context of replication block resolution, however, Pif1 acts in the presence of the replisome, with the CMG helicase present on the leading strand. The presence of the accessory helicase on the nontracking strand is an arrangement that is similar to that for the bacterial replicative hexameric helicase DnaB with its accessory helicases, UvrD or Rep.

**Fig. 6.**
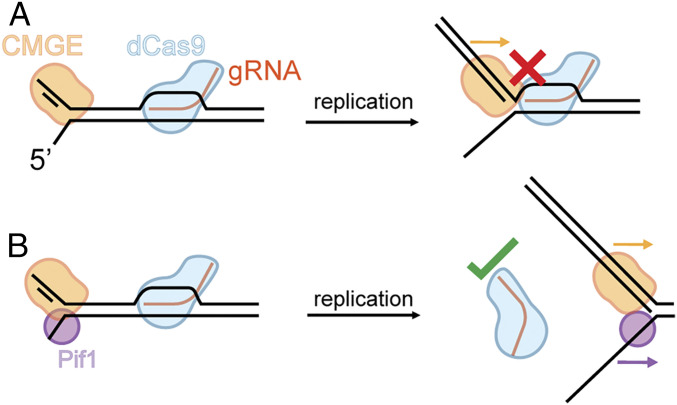
Model of R-loop displacement activity. (*A*) When CMG–Pol ε, which tracks 5′–3′, encounters an R-loop block, it is unable to displace the block. (*B*) With the concerted action of Pif1, the dCas9 R-loop block is cleared. Pif1 is able to fully displace the R-loop, allowing the removal of the dCas9 R-loop as an entity.

We propose a simple mechanism by which Pif1 may facilitate replication bypass of the dCas9 R-loop barrier (Fig. 6). First, we note that helicases that track on ssDNA substrates may also track on one strand of a duplex substrate (44), as exemplified by the CMG motor (45) and by a phage packaging motor (46). Thus, in our model, the 3′–5′ Pif1 helicase functions on the lagging single strand to displace dCas9 R-loops by adding its action to that of CMG. Together, CMG and Pif1 are able to fully displace the block (Fig. 6) in a way that allows the dCas9 complex to rebind to either daughter strand. To bypass a barrier, Pif1 may not need to act in a processive fashion and, thus, might act distributively at the time and place where a replisome is stalled. This is indeed suggested by the protein concentration dependence of Pif1 in replisome bypass of the dCas9 R-loop block.

In summary, we show that Pif1 enables the replisome to bypass a dCas9 R-loop block. Our single-molecule measurements show that both the dCas9 and the R-loop are fully displaced. It has been shown previously that DNA binding by dCas9 is mutagenic in yeast, with mutations clustered near the gRNA target region (47). It is tempting to speculate that Pif1 helps to remove off-target bound dCas9 and that depletion of Pif1 could increase mutagenicity. The dCas9 R-loop complex resembles a stalled RNA polymerase complex, which is also comprised of a protein bound to an R-loop. Our results, therefore, provide information on how Pif1 can help remove stalled RNA polymerases during replication–transcription conflicts. We propose that Pif1, together with the replisome, can support the displacement of both R-loops and protein blocks. We anticipate that this activity could also play a role in the removal of other protein roadblocks outside the context of R-loops.

## Materials and Methods

### Purification of Replisome Components.

CMG helicase (37), Pol ε (38), RFC (48), PCNA (49), Mcm10 (40), MTC (40), Pol δ (39), Pol α (39), Ctf4 (39), and RPA (50) were expressed in *S. cerevisiae* and purified as described previously (37, 39, 40).

For purification of Pif1, a 3XFLAG tag was placed on the N-terminus of the gene encoding Pif1 in the *pRS405 TRP1::GAL* integration vector and integrated into OY001 (*ade2-1 ura3*-*1 his3*-*11*,*15 trp1*-*1 leu2*-*3*,*112 can1*-*100 bar1*Δ *MAT* **a** *pep4*::*KANMX6*). Cells were grown under auxotrophic selection (-Trp) at 30 °C in yeast peptone dextrose (YPD) glucose, then split into 12L YP-glycerol, grown to an OD_600_ of 0.6 at 30 °C, and induced with 2% galactose for 6 h. Cells were harvested by centrifugation at 1,500 × *g*, resuspended with 10 mL of 20 mM Hepes, pH 7.6, 1.2% polyvinylpyrrolidone, and protease inhibitors, and frozen by dripping into liquid nitrogen. Cells were subsequently lysed with a cryogenic grinding mill (SPEX SamplePrep). The lysate was clarified by centrifugation at 19,500 rpm in an SS-34 rotor for 2 h at 4 °C. The supernatant was applied to a 1-mL anti-FLAG M2 column (Sigma) equilibrated with buffer A (350 mM potassium glutamate, 25 mM Hepes, pH 7.5, 10% glycerol, 1 mM dithiothreitol [DTT]) and subsequently washed with 30 column volumes (CV) buffer A. Protein was eluted in buffer A including 200 mg/mL 3xFLAG peptide (EZBiolab). The eluate was dialyzed to 75 mM NaCl for 6 h in a 2-L dialysis buffer (0.1 M NaCl, 25 mM Hepes, 10% glycerol, 1 mM DTT), loaded onto a 0.2-mL Mono-S column (GE healthcare) and eluted with a 10 CV 0.1–0.8 M NaCl gradient in elution buffer (25 mM Hepes, 10% (vol/vol) glycerol, 1 mM DTT). Peak fractions were flash frozen, aliquoted, and stored at −80 °C.

### Purification of dCas9Cys.

The purification of dCas9Cys was adapted from ref. 33. *E. coli* strain Rosetta 2(DE3) containing Addgene plasmid 60815 encoding a dCas9 with single cysteine for site-specific labeling was grown in lysogeny broth medium supplemented with thymine (25 μg/mL) and ampicillin (100 μg/mL) at 37 °C. Upon growth to *A*_600_ = 0.8, the temperature was reduced to 16 °C and protein expression induced by addition of 0.5 mM isopropyl-β-d-thiogalactoside. Cultures were shaken for a further 16 h at 16 °C, then chilled on ice. Cells (7.5 g from 2 L of culture) were harvested by centrifugation, frozen in liquid nitrogen and stored at –80 °C. All subsequent steps were carried out in a cold room maintained at 6 °C. After thawing, cells were resuspended in lysis buffer (20 mM Tris⋅HCl pH 7.6, 0.1 mM ethylenediaminetetraacetic acid [EDTA], 1 mM DTT, 150 mM NaCl, 5% [vol/vol] glycerol), and 2× protease inhibitor mixture tablets and 0.7 mM phenylmethylsulfonyl fluoride were added to inhibit proteolysis. Cells were lysed by being passed twice through a French press (12,000 psi), and cell debris were then removed by centrifugation. Crude supernatant (68 mL) was brought to 0.3% (vol/vol) in polyethylenimine (PEI) and vigorously stirred. After 50 min, the white precipitate was separated by centrifugation. The remaining pellet was homogenized by stirring in lysis buffer for 15 min. The remaining white precipitate was immediately collected by centrifugation and the supernatant discarded. The remaining pellet was further homogenized in lysis buffer +350 mM NaCl for 15 min. After centrifugation, the supernatant containing 10XHis–MBP–dCas9-dL5 was collected yielding fraction I (62 mL). Proteins that were precipitated from fraction I by the addition of solid ammonium sulfate (0.4 g/mL) by stirring for 60 min, and subsequently collected by centrifugation and dissolved in 30 mL of HisTrap buffer (50 mM Tris⋅HCl pH 7.6, 0.5 mM EDTA, 1 mM DTT, 20 mM Imidazole, 500 mM NaCl, 5% [vol/vol] glycerol). The solution was dialyzed against 2 L of the same buffer overnight, to yield fraction II. Fraction II was loaded onto an equilibrated HisTrap column. 10XHis–MBP–TEV–dCas9 was eluted using a linear gradient 20–300 mM imidazole over 20 mL. Pooled fractions were dialyzed against 2 L of TEV buffer (20 mM Hepes pH 7.6, 0.5 mM EDTA, 1 mM DTT, 150 mM NaCl, 10% (vol/vol) glycerol) for 3 h to yield fraction III. Fraction III was then dialyzed against 2 L of TEV buffer containing 1,000 U His-tagged TEV protease (Sigma) for 16 h to cleave the 10XHis–MBP tag. To remove uncleaved 10XHis–MBP–TEV–dCas9, fraction III was loaded onto another HisTrap column. Fractions containing cleaved dCas9 were pooled and dialyzed overnight against of 2 L of HiTrap SP buffer (20 mM Hepes pH 7.6, 0.5 mM EDTA, 2 mM DTT, 150 mM NaCl, 10% (vol/vol) glycerol) to yield fraction IV. Fraction IV was loaded onto an equilibrated 5-mL HiTrap SP column. dCas9 eluted as a single peak. Fractions under the peak were pooled and dialyzed against 2 L of storage buffer (50 mM Hepes pH 7.6, 1 mM EDTA, 3 mM DTT, 300 mM NaCl, 50% [vol/vol] glycerol). Aliquots were frozen in liquid N_2_ and stored at –80 °C.

### Preparation of AF488–dCas9Cys.

Methods described below were adapted from ref. 51. Alexa Fluor 488 (AF488, Invitrogen) was used to label dCas9Cys. First, a total of 2.2 mg of dCas9Cys was reduced with 3 mM Tris(2-carboxyethyl)phosphine (TCEP), pH 7.6 in reducing buffer (50 mM Hepes pH 7.6, 500 mM NaCl, 1 mM EDTA, 70% [wt/vol] ammonium sulfate, 10% [vol/vol] glycerol) at 6 °C for 1 h with gentle rotation to yield fraction I. Fraction I was centrifuged (21,000 × *g*; 15 min) at 6 °C and the supernatant carefully removed. The precipitate was washed with ice cold reducing buffer that had been extensively degassed by sonication and deoxygenated using Ar gas, then pelleted by centrifugation (21,000 × *g*; 15 min) at 6 °C and supernatant removed to yield fraction III. The labeling reaction was carried out on fraction III, now devoid of reducing agent, using 10-fold molar excess of maleimide conjugated AF488 with 22 μM dCas9Cys in 600 μL of deoxygenated and degassed labeling buffer (50 mM Hepes, pH 7.6, 500 mM NaCl, 1 mM EDTA, 10% [vol/vol] glycerol). The reaction was allowed to proceed for 3 h at 23 °C, followed by further incubation at 6 °C overnight with gentle rotation (in the dark). The reaction was subsequently quenched using 3 mM TCEP for 1 h at 6 °C yielding fraction IV. Fraction IV was applied at 1 mL/min to a column (1.5 × 10 cm) of Superdex G-25 resin equilibrated with filtration buffer (50 mM Hepes pH 7.6, 3 mM DTT, 1 mM EDTA, 300 mM NaCl, 10% [vol/vol] glycerol). Fractions containing the labeled dCas9Cys were pooled and dialyzed into storage buffer (50 mM Tris⋅HCl pH 7.6, 1 mM EDTA, 500 mM NaCl, 25% [vol/vol] glycerol). Labeled AF488–dCas9 was frozen in liquid N_2_ and stored in aliquots at –80 °C. The degree of labeling was measured to be 1 fluorophore per dCas9 by ultraviolet/visible spectrophotometry.

### Ensemble DNA Replication Assays.

Radionucleotides were purchased from PerkinElmer, and unlabeled nucleotides were from GE Healthcare. DNA oligonucleotides were purchased from IDT. Replication assays were performed as previously described (52, 35). Reaction volumes were 25 μL and contained 1.5 nM of 2.8-kb dsDNA template ligated to a synthetic fork annealed to a 5′-^32^P labeled 37-mer DNA primer. Except where otherwise noted, reactions were loaded with 30 nM CMG, 20 nM dCas9, 60 nM gRNA, and, when indicated, 80 nM Mcm10, for 5 min at 30 °C, followed by incubation with 5 nM RFC, 25 nM PCNA, 20 nM Pol delta, and 120 μM dNTPs for 5 min. The gRNA targeting dCas9 to either the leading strand at 0.6 kb or the lagging strand at 1.0 kb is fully described in ref. 33. Unless otherwise indicated, reactions were initiated with 5 mM ATP and 600 nM RPA and allowed to replicate for 10 min, followed by the addition of the indicated concentration of Pif1 and/or Pif1 storage buffer for balancing salt concentration. Reactions were stopped at either 20 min or the indicated timepoints with the addition of 2× STOP buffer (40 mM EDTA and 1% SDS [wt/vol]). Reactions were analyzed on 1.3% (wt/vol) alkaline agarose gels for 17 h, backed with DE81 paper, and compressed. Gels were exposed to a phosphoimager screen and imaged with a Typhoon FLA 9500 (GE Healthcare).

### Linear Forked Doubly Tethered DNA Substrates.

Linear DNA substrates were constructed as described previously without modification (33, 41, 53).

### Flow Cell Preparation.

Flow cells were prepared as described previously (41, 54, 55). Briefly, a polydimethylsiloxane (Sylgard) lid was placed on top of a polyethyleneglycol-biotin-functionalized microscope slide (24 × 24 mm, Marienfeld) to create a 1-mm-wide and 100-μm-high flow channel (∼1 μL volume). Polyethylene tubes (PE-60: 0.76-mm inlet diameter and 1.22-mm outer diameter, Walker Scientific) were inserted to allow for a buffer flow. To help prevent nonspecific interactions of proteins and DNA with the surface, the chamber was blocked with blocking buffer (50 mM Tris⋅HCl pH 7.6, 50 mM KCl, 2% [vol/vol] Tween-20).

For reactions containing the dCas9–gRNA block, the forked DNA substrates were first incubated with 450 nM gRNA (Integrated DNA Technologies) and 240 nM AF488–dCas9 in the presence of 100 μg/mL heparin (Sigma) (33). Excess dCas9–gRNA and heparin were removed by elution from a 0.8-mL Sepharose 4B (Sigma) spin column (Sigma) equilibrated in replication buffer (25 mM Tris⋅HCl, pH 7.6, 10 mM magnesium acetate, 50 mM potassium glutamate, 40 μg/mL bovine serum albumin, 0.1 mM EDTA, 5 mM DTT, and 0.0025% [vol/vol] Tween-20) + 300 mM sodium chloride. The forked DNA substrates (20 pM) were flowed through the chamber for 20 min at 17 μL/min in the presence of 200 μM chloroquine (Sigma). The DNA was visualized by flowing in replication buffer containing 150 nM Sytox Orange (Life Technologies).

### Single-Molecule Replication Reactions.

Single-molecule replication assays were carried out as described previously (41). Briefly, 30 nM CMG was loaded at 10 μL/min in CMG loading buffer, with 60 nM Mcm10 and 400 μM ATP. Then, replication reactions were initiated by introducing the replication mix, containing 60 nM Mcm10, 20 nM Pol ε, 20 nM Pol δ, 20 nM Pol α, 20 nM Ctf4, 20 nM PCNA, 200 nM RPA, and 30 nM MTC in replication buffer supplemented with 5 mM ATP, 125 μM dCTP, dGTP, dATP, and dTTP, and 250 μM CTP, GTP, ATP, and UTP, and 150 nM Sytox Orange. In reactions with Pif1, the replication mix with 100 nM Pif1 was introduced after 15 min.

### Imaging of Single-Molecule Reactions.

All single-molecule assays were carried out on an inverted microscope (Nikon Eclipse Ti-E) fitted with a CFI Apo TIRF 100× oil-immersion objective (N.A. 1.49, Nikon). The temperature was maintained at 31.2 °C by an electrically heated chamber (Okolab). dsDNA was visualized every 10 s for 30 min by exciting the Sytox Orange. with a 568-nm laser (Coherent, Sapphire 568–200 CW) at 80 mW/cm^2^. The red fluorescently labeled gRNA was excited at 80 mW/cm^2^ with a 647-nm laser (Coherent, Obis 647–100 CW). The AF488–dCas9 was visualized with a 488-nm laser (Coherent, Sapphire 488–200 CW) at 140 mW/cm^2^. The signals were spectrally separated using appropriate filter sets (Chroma) and fluorescence signals collected on an Evolve 512 Delta EMCCD (Photometics). Typically, nine fields of view were selected for imaging. Single-molecule experimental results were derived from at least three or four technical replicates for each experimental condition.

### Analysis of Single-Molecule Replication Events.

All analyses were carried out using ImageJ/Fiji (1.51w), Matlab 2016b, and in-house built plugins. To obtain the product length for every replication event, we tracked the position and the integrated intensity of the leading-strand spot over time. We measured the average position of the dCas9–gRNA relative to the length of the DNA substrate.

### Quantification and Statistical Analysis.

The number of molecules or events analyzed is indicated in the text or figure legends. Errors reported in this study represent the SEM, SD, or the error of the fit, as indicated in the text or figure legends. Every single-molecule replication experiment was carried out at least in triplicate.

## Supplementary Material

Supplementary File

## Data Availability

All study data are included in the article and supporting information.

## Change History

December 1, 2020: The author line has been updated.
