# Supplementary material for: Replisome bypass of a protein-based R-loop block by Pif1
Source: Proc Natl Acad Sci U S A. 2020 Nov 16;117(48):30354–61. doi: 10.1073/pnas.2020189117 (PMC7720201; doi:10.1073/pnas.2020189117)
Supplement: Supplementary File [file pnas.2020189117.sapp.pdf]

PNAS
www.pnas.org

## Supplementary Information for

# Replisome bypass of a protein-based R-loop block by Pif1

Grant D. Schauer[a,1,*], Lisanne M. Spenkelink[b,c,1], Jacob S. Lewis[b,c,e], Olga Yurieva[d], Stefan H. Mueller[b,c], Antoine M. van Oijen[b,c],* and Michael E. O'Donnell[d]*

*Correspondence should be addressed to:

Grant D. Schauer, grant.schauer@colostate.edu

Antoine M. van Oijen, Email: vanoijen@uow.edu.au

Michael E. O'Donnell, Email: odonnel@rockefeller.edu

**This PDF file includes:**

Figures S1 to S4

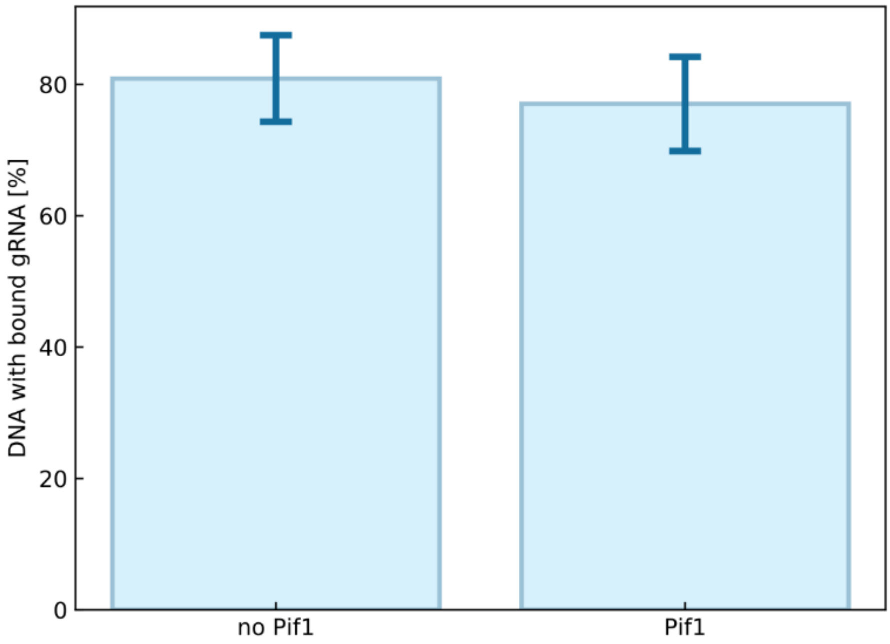


**Fig. S1. Pif1 does not displace roadblocks in absence of the replisome**. Results are from single-molecule replication studies (see Methods). The amount of DNA molecules with bound dCas9-gRNA was observed before addition of Pif1 and after 30 min of incubation with 80 nM Pif1. Over 4 individual movies the amount of bound dCas9-gRNA stayed constant within uncertainty levels. (80.9 ± 6.6)% in absence, (77.0 ± 7.2)% in presence of Pif1.

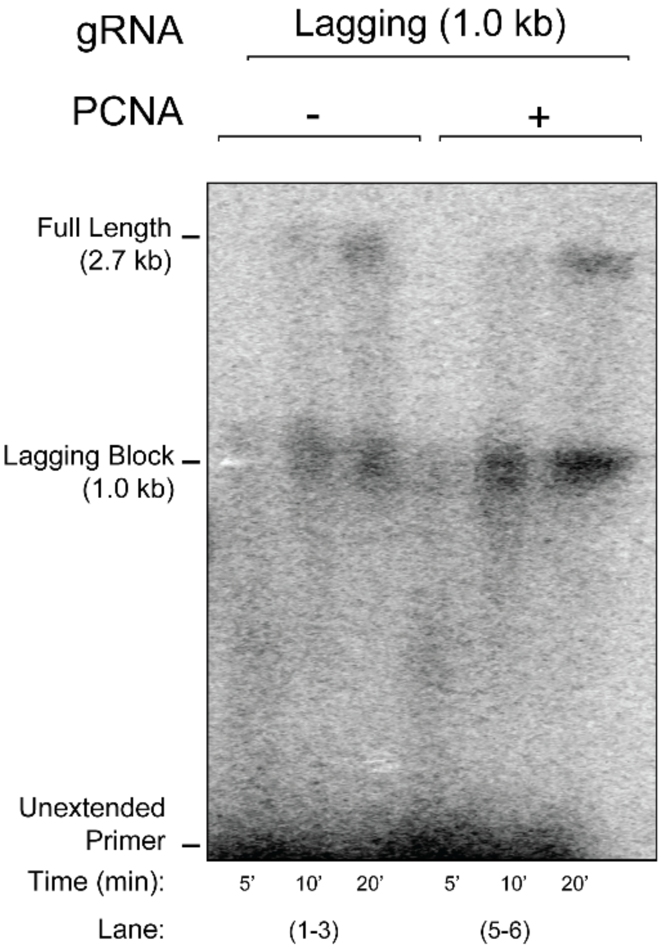


**Fig. S2. PCNA is not required for Pif1 mediated bypass of a lagging strand dCas9 block.** Alkaline agarose gel of the indicated replication products in the presence of a lagging strand block.

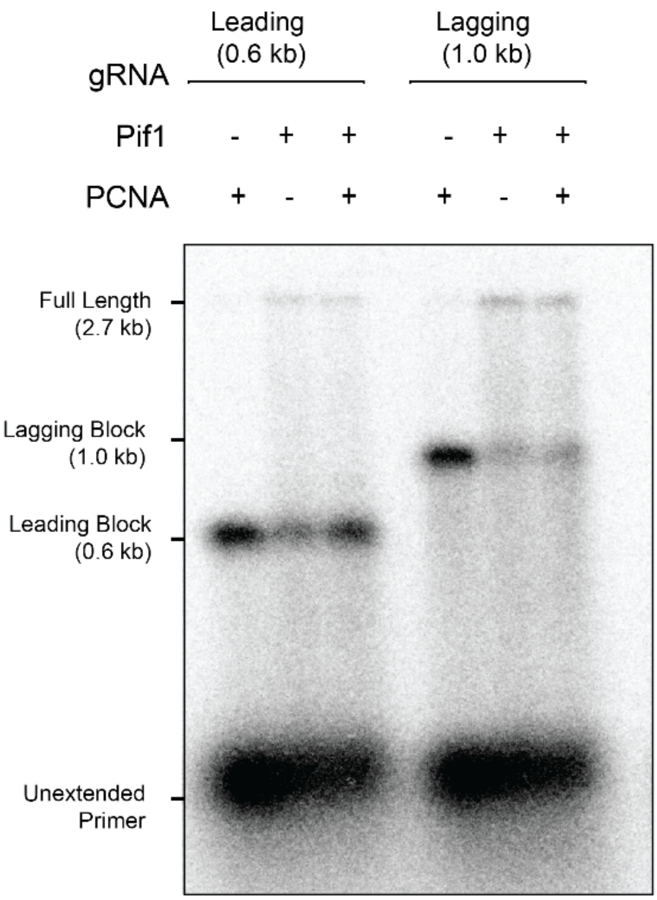


**Fig. S3. PCNA does not enhance bypass in the presence of the MTC complex and Mcmc10**. Replication products in the presence of the indicated block and the presence or absence of Pif1 and/or PCNA is shown. MTC and Mcm10 were present in all reactions.

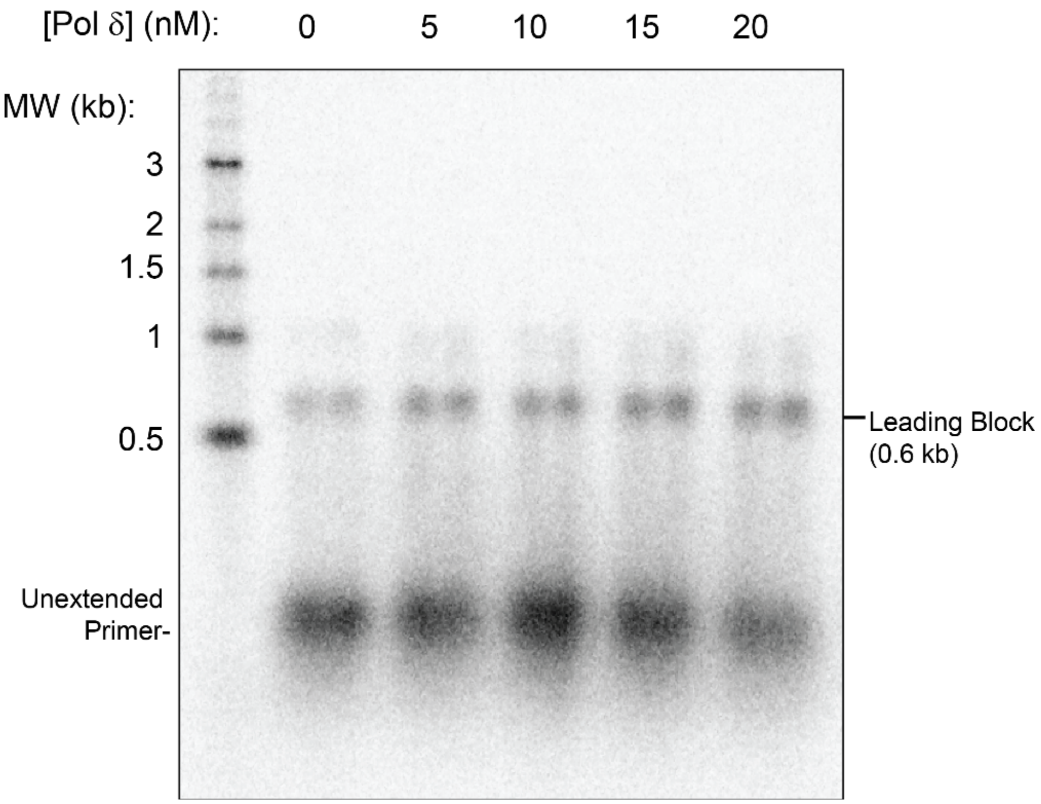


**Fig. S4. Addition of Pol delta-PCNA and Pol delta does not permit displacement of the dCas9 R-loop block**. Replication products in the presence of the leading strand dCas9 R-loop block at 0.6 kb are shown. In addition to CMG, Pol delta, RFC, PCNA, Mcm10, and MTC, Pol [illegible] were present at 20 nM, and Pol delta was added at the indicated concentrations.
